# Reproducibility of Search Strategies Is Poor in Systematic Reviews Published in High-Impact Pediatrics, Cardiology and Surgery Journals: A Cross-Sectional Study

**DOI:** 10.1371/journal.pone.0163309

**Published:** 2016-09-26

**Authors:** Jonathan B. Koffel, Melissa L. Rethlefsen

**Affiliations:** 1 Bio-Medical Library, University of Minnesota, Minneapolis, Minnesota, United States of America; 2 Spencer S. Eccles Health Sciences Library, University of Utah, Salt Lake City, Utah, United States of America; McGill University, CANADA

## Abstract

**Background:**

A high-quality search strategy is considered an essential component of systematic reviews but many do not contain reproducible search strategies. It is unclear if low reproducibility spans medical disciplines, is affected by librarian/search specialist involvement or has improved with increased awareness of reporting guidelines.

**Objectives:**

To examine the reporting of search strategies in systematic reviews published in Pediatrics, Surgery or Cardiology journals in 2012 and determine rates and predictors of including a reproducible search strategy.

**Methods:**

We identified all systematic reviews published in 2012 in the ten highest impact factor journals in Pediatrics, Surgery and Cardiology. Each search strategy was coded to indicate what elements were reported and whether the overall search was reproducible. Reporting and reproducibility rates were compared across disciplines and we measured the influence of librarian/search specialist involvement, discipline or endorsement of a reporting guideline on search reproducibility.

**Results:**

272 articles from 25 journals were included. Reporting of search elements ranged widely from 91% of articles naming search terms to 33% providing a full search strategy and 22% indicating the date the search was executed. Only 22% of articles provided at least one reproducible search strategy and 13% provided a reproducible strategy for all databases searched in the article. Librarians or search specialists were reported as involved in 17% of articles. There were strong disciplinary differences on the reporting of search elements. In the multivariable analysis, only discipline (Pediatrics) was a significant predictor of the inclusion of a reproducible search strategy.

**Conclusions:**

Despite recommendations to report full, reproducible search strategies, many articles still do not. In addition, authors often report a single strategy as covering all databases searched, further decreasing reproducibility. Further research is needed to determine how disciplinary culture may encourage reproducibility and the role that journal editors and peer reviewers could play.

## Introduction

One of the defining attributes of systematic reviews is their use of explicit and reproducible methods to gather, appraise, and summarize the best available evidence. Full reporting of the methods used helps the reader to assess the strength and comprehensiveness of the review and makes it easier to update and expand on the original systematic review as new evidence is published [1].

Despite the need for transparent reporting of systematic review methods, many reviews have been found to contain incomplete reporting [2–16]. Reporting guidelines such as PRISMA and MOOSE have existed for many years and been widely shared to help guide authors on what elements need to be reported and how best to report them [17, 18]. As of August 2016, over 175 journals and publishers have formally endorsed the PRISMA Statement [19] with many others including them in instructions to authors. Despite the existence and promulgation of these guidelines, reporting quality is still suboptimal [2, 7, 20] and it is unclear if endorsement of guidelines leads to better reporting [5, 21–23].

A particular area of concern is the reporting of search strategies. A comprehensive search forms the foundation of any systematic review since it gathers the articles that will be appraised and summarized. Complete reporting of the search strategy allows the reader to assess if the authors chose the appropriate databases, terms, and limits for the question they are answering. The authors of the PRISMA Statement considered the search strategy “an essential part of the report of any systematic review” and recommended that authors “[p]resent the full electronic search strategy for at least one major database, including any limits used, such that it could be repeated” [1]. Other guidelines provide similar recommendations [18, 24–26].

Previous studies have found that some elements, such as names of databases searched, are well-reported, but one of the most important elements, the inclusion of a reproducible search strategy, was often missing or incomplete [8, 27–33]. There is emerging evidence that librarian involvement in a systematic review may result in better search strategy reporting, including inclusion of a reproducible search strategy [28, 30, 31]. It is also plausible that author awareness of best practices for reporting could result in increased reporting of reproducible searches, though this has not yet been shown.

Existing research on the reporting of reproducible searches has been limited in several important ways. First, much of the research examined articles from before the introduction of PRISMA. PRISMA is perhaps the best known and most widely used reporting guideline [34] and has the most explicit criteria for reporting of search strategies, thus its adoption could be a tipping point for reporting quality. Previous research into the effect of reporting guidelines on reporting quality has not shown a strong effect of reporting guidelines on search strategy reporting and reproducibility [5, 21–23, 35]. These studies, however, have treated the search strategy as a single entity rather than looking critically at specific aspects of the search strategy reporting, such as the inclusion of a reproducible search. Second, authors of previous studies have used a range of definitions for what constitutes a reproducible search. Finally, most research has focused on General/Internal Medicine journals and little is known about recent reporting trends in other medical disciplines.

In this study, we examine the reproducibility of systematic reviews published in high-impact Pediatrics, Surgery and Cardiology journals. We selected these disciplines since they have large numbers of specialty journals and allow us to expand on the findings on reproducibility in General/Internal Medicine journals from previous studies. We look at rates of overall reproducibility as well as reporting of elements such as databases, dates, and search terms that contribute to reproducibility. Finally, we investigate whether endorsement of reporting guidelines, librarian involvement or other factors are associated with reporting of reproducible searches.

## Methods

### Article Identification and Search Extraction

We first identified the ten Pediatrics, Surgery and Cardiology journals in each discipline with the highest impact factors according to the 2012 Journal Citation Reports (impact factors retrieved on August 22, 2013) [36]. One author (JK) examined the instructions to authors for each journal in November, 2013 and recorded whether they mentioned a systematic review reporting guideline. When possible, the Wayback Machine [37] was used to examine the instructions to authors that were available in the first 6 months of 2012 and these were compared with the instructions available in November, 2013.

One author (J.K.) conducted a search in PubMed on September 2, 2013 using a modified version of search hedge created by Montori et al. [38] *((search[Title/Abstract] OR meta-analysis[Publication Type] OR MEDLINE[Title/Abstract] OR EMBASE[Title/Abstract] OR Meta-analysis[Title/Abstract] OR (systematic[Title/Abstract] AND review[Title/Abstract])*) to identify all systematic reviews published in these journals which were added to PubMed between January and December of 2012. To be included in the study, the article had to indicate that at least one published literature database was searched, pre-specify the inclusion and exclusion criteria for the study, and could not limit to a certain number of journals or subset of journals. The study could include a meta-analysis or be published without quantitative analysis. We excluded other study types (e.g., randomized controlled trials, case studies, etc) and general literature reviews not meeting our criteria. We independently reviewed the abstract and full-text (if the abstract was unclear) of retrieved articles to separate the systematic reviews from other publication types. Abstracts and full-text were reviewed in a single round. Disagreements were resolved by discussion.

Systematic review reporting standards [1, 18, 24–26], previous research [28, 29, 33] and the authors’ personal knowledge and experience were used to generate a list of elements necessary for complete search reporting. We independently extracted information on each element from all eligible articles. Disagreements were resolved by discussion.

For relevant search reporting elements (e.g., first year searched, specific search terms listed, limits indicated), we recorded whether that element was reported for each database searched. For ease of analysis, in these cases the results were collapsed down to indicate if the element was reported for any of the databases or for all of the databases (henceforth “any/all”).

Finally, we recorded whether or not the authors listed the provider or interface for the database (e.g., searched MEDLINE using the Ovid interface) since the interface can affect how the search was conducted. For some databases, however, there is only one interface for the database (e.g., Scopus) or the name of the provider was used in lieu of the database name (e.g., PubMed). For each database reported in an included article, we recorded if the provider was explicitly named and/or could be inferred.

### Analyses

We began by examining how often each search element was reported across all systematic reviews in the sample and in each discipline. In addition, we determined whether or not the article contained one or more reproducible search strategies. Different authors have defined a reproducible search in different ways [28, 32, 33, 39]. For this study, we chose a definition close to that required by the Cochrane Collaboration [24]. For the purposes of this study, a reproducible search must indicate the database searched, the first and last years searched, whether limits were applied to the search (either as stated in the text or apparent from the search strategy), and provide a complete search strategy (e.g., exact search terms and the Boolean logic to connect them).

A global chi-square test was used to examine differences by discipline in how often different search elements were reported and how often a reproducible search strategy was provided. When the global chi-square test was significant, post-hoc tests were run to determine which groups were significantly different.

Logistic regression was used to investigate the association between inclusion of a reproducible search for any or all of the mentioned databases and endorsement of a systematic review reporting guideline by the publishing journal, author mention of a reporting guideline, author use of a PRISMA flowchart (considered a proxy for awareness of PRISMA), librarian/information specialist involvement, or medical discipline. Predictors around reporting guidelines (e.g., PRISMA or MOOSE) were chosen since the guidelines require reporting of full search strategies. Librarian/information specialist involvement was selected since previous research has found that librarian involvement improves the search quality and reporting of search strategies [28, 31]. Medical discipline was chosen in order to examine if disciplinary culture and precedent may contribute to reporting of reproducible searches.

We first conducted a set of bivariate logistic regressions to assess the association (odds ratio and 95% confidence interval) between each individual predictor and inclusion of a reproducible search for either a single database or all databases used in the systematic review. We then conducted a multivariable logistic regression, entering all predictors into a single model and again examining association between each predictor and inclusion of a reproducible search for either a single database or all databases used in the systematic review. This multivariable approach allowed us to examine the independent association of each variable, controlling for the others. As there were three disciplines included in our study, in our regression analyses, we set one discipline as the reference (Surgery) and compared the other two disciplines (Cardiology and Pediatrics) against it. Surgery was chosen as the reference category since it contained largest proportion of the included articles.

Model fit for the logistic regression models was assessed using the C-statistic (above .7 considered reasonable) and Hosmer-Lemeshow goodness-of-fit test (p>.05 indicating acceptable fit). We examined the predictor variables for multicollinearity by entering them into a linear regression model and calculating the tolerance of each (values above .2 considered acceptable).

A significance level of p < .05 was chosen for all analyses. A large number of pre-specified analyses were conducted, but no explicit adjustments were made for multiple comparisons due to the exploratory nature of the study.

## Results

**Fig 1:**
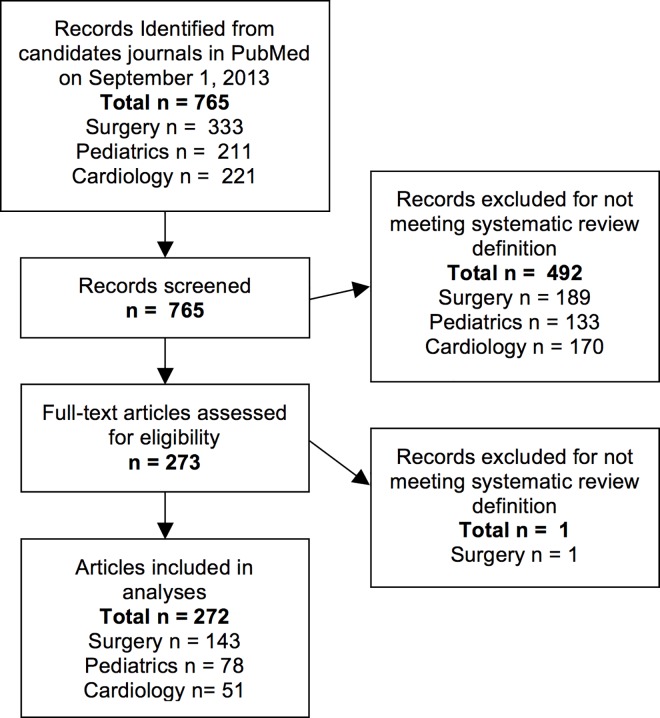
Flow Chart of Included Studies.

We searched PubMed on September 1, 2013 and retrieved 765 articles which were added to PubMed between January and December of 2012 from the candidate journals. We independently reviewed the abstract and full-text of each article and identified 272 systematic reviews (available at http://z.umn.edu/15qt) (Fig 1) published in 25 journals which met our inclusion criteria (Table 1). No systematic reviews could be identified from 2012 in 5 of the 30 original candidate journals. Only 12/25 journals (48%) identified or endorsed a reporting guideline in their instructions to authors. We were able to identify instructions to authors for 11 of the candidate journals using the Wayback Machine (9 of the 25 with included articles). In one case (American Journal of Transplantation), the instructions to authors were from 2011 and thus the Wayback Machine was not consulted. While in most cases the review of instructions to authors from November 2013 and the Wayback Machine agreed, in one case (Archives of Surgery/JAMA Surgery), we discovered that instructions to follow PRISMA/MOOSE reporting guidelines were added in 2013. In this case, we counted JAMA Surgery as not endorsing a reporting guideline in 2012.

**Table 1:** Included Journals.

	Included Articles	Endorsed SR Guideline ‡	*Wayback Machine* Verified
**Surgery Journals (n = 143)**			
American Journal of Surgical Pathology	0		
American Journal of Transplantation	4		#
Annals of Surgery	26	■	
Annals of Surgical Oncology	32		⭕
Archives of Surgery (JAMA Surgery)	7		⭕
British Journal of Surgery	44	■	⭕
Endoscopy	4		
Journal of the American College of Surgeons	7		
Journal of Neurology, Neurosurgery and Psychiatry	15	■	
Surgery for Obesity and Related Diseases	4	■	
**Pediatrics Journals (n = 78)**			
Archive of Diseases in Childhood	11	■	
Archive of Diseases in Childhood: Fetal & Neonatal Edition	1	■	
Archives of Pediatric and Adolescent Medicine (JAMA Pediatrics)	6		
European Child and Adolescent Psychiatry	4		
Journal of the American Academy of Child and Adolescent Psychiatry	7		⭕
Journal of Pediatrics	9		
Pediatric Allergy and Immunology	3		⭕
Pediatric Infectious Disease Journal	7		
Pediatrics	30	■	
Seminars in Fetal and Neonatal Medicine	0		
**Cardiology Journals (n = 51)**			
Circulation: Cardiovascular Interventions	3	■	⭕
Circulation: Cardiovascular Genetics	0	■	⭕
Circulation: Heart Failure	5	■	⭕
Circulation Research	1		⭕
Circulation	7	■	⭕
European Heart Journal	16	■	⭕
Journal of the American College of Cardiology	15		
JACC: Cardiovascular Imaging	2		
JACC: Cardiovascular Interventions	2		
Nature Reviews Cardiology	0		⭕

SR = systematic review.

‡ Journal either formally endorsed the PRISMA Statement or mentioned a SR reporting guideline in their instructions to authors.

# Author instructions in 2013 were dated 2011, so Wayback Machine was not consulted.

### Search Reporting Elements

No search reporting element was reported in all articles (Table 2). Rates of reporting the search elements for *at least one* database in the systematic review ranged from 91% for indication of search terms down to 22% for indicating when the search was executed and 33% for providing a full search strategy. Rates of reporting the search elements for *all* databases in the systematic review (S1 Table) were lower in most cases, with some elements being markedly lower. Of systematic reviews that searched more than one database, a minority provided search strategies or dates searched for a specific database (Table 2), instead providing terms and dates that applied to all databases searched in the systematic review. There were significant differences between disciplines on reporting several search elements, including inclusion of a full search strategy, database years searched and date search executed (Table 2).

**Table 2:** Reporting of Search Strategy Elements For One or More Databases n (%).

	Surgery	Pediatrics	Cardiology	Total
**Core Search Elements**				
Named Database Provider	88 *(62)*	50 *(64)*	28 *(55)*	166 *(61)*
Named/Inferred Database Provider	105 *(73)*	57 *(73)*	34 *(67)*	196 *(72)*
Specific Year Given for First Date Searched	91 *(64)*	51 *(65)*	22 *(43)*	164 *(60)* ‡* §*
Specific Date Given for Last Date Searched •	106 *(74)*	52 *(67)*	37 *(73)*	195 *(72)*
Indicated Date Search Was Executed	34 *(24)*	16 *(21)*	11 *(22)*	61 *(22)*
Indicated Date Search Was Updated	1 *(1)*	6 *(8)*	0 *(0)*	7 *(3)* †* ‡*
Provided Specific Search Terms	135 *(94)*	66 *(84)*	47 *(92)*	248 *(91)* †*
Provided a Full Search Strategy	29 *(20)*	42 *(54)*	18 *(35)*	89 *(33)* †*** ‡* §*
Indicated If Limits Were Used	104 *(73)*	60 *(77)*	39 *(77)*	203 *(75)*
**Search Elements Indicated for a Specific Database** #				
Search Terms	24 *(20)*	22 *(32)*	11 *(24)*	57 *(25)*
Start/End Dates	21 *(18)*	28 *(41)*	8 *(18)*	57 *(25)* †** ‡**
Full Search Strategy	16 *(13)*	32 *(47)*	9 *(20)*	56 *(24)* †**
Limits	7 *(6)*	17 *(25)*	11 *(24)*	35 *(15)* †** §**
**Reproducibility**				
Any Search Strategy was Reproducible $	16 *(11)*	33 *(42)*	10 *(20)*	59 *(22)* †*** ‡**
All Search Strategies were Reproducible $ #	9 *(8)*	19 *(28)*	4 *(9)*	31 *(14)* †*** ‡*

† Pediatrics vs. Surgery

‡ Pediatrics vs. Cardiology

§ Surgery vs. Cardiology

*p < .05,

** p < .01

***p < .001.

• Specific month and year.

$ Article indicates database searched, the first and last years searched, whether limits were applied to the search, and provide a complete search strategy.

# Limited to articles which searched more than one database.

The majority (75%) of articles reported whether or not limits were applied (Table 3). Of those that reported limit use, the most popular type was no limit use (32%), followed by limiting to English language (25%) and human (19%) articles.

**Table 3:** Use of Search Limits n (%).

	Surgery	Pediatrics	Cardiology	Total
Indication of Limit Use	104 *(73)*	60 *(77)*	39 *(76)*	203 *(75)*
No Limits Used	50 *(35)*	22 *(28)*	16 *(31)*	88 *(32)*
Limited to Human	23 *(16)*	14 *(18)*	15 *(29)*	52 *(19)* §*
Limited to English Articles	35 *(25)*	22 *(28)*	12 *(24)*	69 *(25)*
Limited to English and Other Languages	6 *(4)*	4 *(5)*	0 *(0)*	10 *(4)*
Limited by Publication Type	8 *(6)*	9 *(12)*	12 *(24)*	29 *(11)* §***

§ Surgery vs. Cardiology

*p < .05

** p < .01

***p < .001.

The majority of authors (85%) used methods other than traditional database searching to find potential articles (Table 4). These included searching the references of included articles (77%) and non-included articles (50%) such as other review articles. Fewer authors (28%), however, used grey literature searching methods to identify unpublished or nontraditional sources of evidence. Of these, the most popular grey literature sources included conference abstracts (14%), clinical trial registries (7%), and contacting authors of included articles or similar articles (6% and 5%, respectively).

**Table 4:** Additional Search Methods n (%).

	Surgery	Pediatrics	Cardiology	Total
References of Included Articles	112 *(78)*	61 *(78)*	36 *(71)*	209 *(77)*
References Non-included Articles	79 *(55)*	35 *(45)*	21 *(41)*	135 *(50)*
Conference Abstracts Reviewed	13 *(9)*	13 *(17)*	11 *(22)*	37 *(14)* §*
ClinicalTrials.gov	8 *(6)*	5 *(6)*	7 *(14)*	20 *(7)*
Other Clinical Trial Registry	14 *(10)*	5 *(6)*	1 *(2)*	20 *(7)*
Prominent Authors Contacted	6 *(4)*	8 *(10)*	3 *(6)*	17 *(6)*
Handsearching of Journals	8 *(6)*	3 *(4)*	2 *(4)*	13 *(5)*
Authors of Included Articles Contacted	9 *(6)*	3 *(4)*	1 *(2)*	13 *(5)*
Personal Libraries of Authors Reviewed	5 *(4)*	1 *(1)*	0 *(0)*	6 *(2)*
FDA Contacted	2 *(1)*	1 *(1)*	2 *(4)*	5 *(2)*
Websearch (e.g. Google)	3 *(2)*	1 *(1)*	2 *(4)*	5 *(2)*
Drug/Instrument Manufacturers Contacted	3 *(2)*	1 *(1)*	0 *(0)*	4 *(2)*

§ Surgery vs. Cardiology

*p < .05

** p < .01

***p < .001.

### Searchers and Reporting Guidelines

Just over a quarter of authors (28%) reported who designed or executed the search strategy (Table 5). Librarian or search specialist involvement could only be identified in 17% of articles. For both of these variables, however, there were strong disciplinary differences. Of those that mentioned who performed the search, most indicated that a librarian or physician (46% and 38%, respectively) contributed. When librarians or search specialists were involved in an article, most often they were executing the search (80%) or advising on search design (13%).

**Table 5:** Searchers and Standards n (%).

	Surgery	Pediatrics	Cardiology	Total
Reporting/Conduct Standard Mentioned	91 *(64)*	36 *(44)*	25 *(49)*	152 *(55)* †**
- PRISMA	61 *(43)*	14 *(18)*	16 *(31)*	91 *(34)* †***
- MOOSE	18 *(13)*	13 *(17)*	8 *(16)*	39 *(14)*
- Cochrane	17 *(12)*	4 *(5)*	4 *(8)*	25 *(9)*
- Multiple/Other	12 *(8)*	10 *(13)*	3 *(6)*	25 *(9)*
PRISMA Flowchart Included	123 *(86)*	61 *(79)*	46 *(90)*	230 *(85)*
Librarian/Search Specialist Mentioned	19 *(13)*	26 *(33)*	0 *(0)*	45 *(17)* †*** ‡*** §**
Indication of Who Conducted the Search	39 *(27)*	35 *(45)*	3 *(6)*	177 *(28)* †*** ‡** §**
**Profession of the Searcher(s) (if indicated)**				
Physician	16 *(41)*	11 *(31)*	2 *(67)*	29 *(38)*
Nurse	0 *(0)*	0 *(0)*	0 *(0)*	0 *(0)*
Public Health	0 *(0)*	2 (*6)*	0 *(0)*	2 *(3)*
Librarian	14 *(36)*	21 *(60)*	0 *(0)*	36 *(46)*
Information Specialist	1 *(3)*	2 *(6)*	0 *(0)*	3 *(4)*
SR Specialist	0 *(0)*	1 *(3)*	0 *(0)*	1 *(1)*
PhD	2 *(5)*	4 *(11)*	0 *(0)*	6 *(8)*
Other	2 *(5)*	5 *(14)*	2 *(67)*	9 *(12)*
**Librarian Role (if mentioned)**				
Librarian Executed Search	15 *(79)*	21 *(81)*	n/a	36 *(80)*
Librarian Advised on Search	2 *(11)*	4 *(15)*	n/a	6 *(13)*
Librarian Reviewed Search	2 *(11)*	0 *(0)*	n/a	2 *(4)*
Librarian Helped Write	0 *(0)*	2 *(8)*	n/a	2 *(4)*
Librarian Helped Revise	0 *(0)*	2 *(8)*	n/a	2 *(4)*
Librarian Helped with Statistics	0 *(0)*	1 *(4)*	n/a	1 *(2)*

† Pediatrics vs. Surgery

‡ Pediatrics vs. Cardiology

§ Surgery vs. Cardiology

*p < .05

** p < .01

***p < .001.

Half of the included articles (49%) indicated the use of some reporting guideline, most commonly the PRISMA Statement (34% of all articles) (Table 5).

### Reproducibility

Overall, 22% of articles provided at least one reproducible search strategy and 13% provided a reproducible strategy for all databases searched in the article (Table 6). Table 7 indicates the frequency of predictor variables (other than journal endorsement of a reporting guideline and discipline, which are presented in Table 1) by journal.

**Table 6:** Predictors for Inclusion of Reproducible Search Strategy.

	Any Search Reproducible Odds Ratio (95% CI)		All Searches Reproducible # Odds Ratio (95% CI)	
	Bivariate	Multivariable	Bivariate	Multivariable
Reporting/Conduct Standard Mentioned	1.14 (.64–2.04)	1.38 (.72–2.65)	.96 (.47–2.12)	1.20 (.55–2.59)
Librarian/Information Specialist Involved	**3.02 (1.52–6.00)**	2.04 (.93–4.47)	2.74 (1.23–6.10)	1.92 (.80–4.64)
PRISMA Diagram Included	1.70 (.68–4.27)	2.08 (.76–5.68)	1.08 (.39–2.97)	1.26 (.43–3.70)
Journal Endorsed Reporting Guideline	1.07 (.50–2.33)	1.04 (.44–2.47)	1.80 (.42–2.79)	1.16 (.43–3.15)
Cardiology vs. Surgery	1.94 (.82–4.60)	2.26 (.93–5.53)	1.45 (.47–4.45)	1.66 (.53–5.24)
Pediatrics vs. Surgery	**5.82 (2.03–11.58)**	**5.80 ((2.77–12.14)**	**4.59 (2.02–10.4)**	**4.39 (1.85–10.43)**

Bolded cells indicate significant results (p < .05).

# Limited to articles which searched more than one database.

**Table 7:** Predictor Variables by Journal.

	N	Reproducible Search † n (%)	PRISMA Diagram Included n (%)	Reporting Guideline Mentioned n (%)	Librarian/IS Involved n (%)
American Journal of Transplantation	4	0 (0)	4 (100)	3 (75)	1 (25)
Annals of Surgery	26	3 (12)	22 (85)	16 (62)	4 (15)
Annals of Surgical Oncology	32	4 (13)	25 (78)	19 (59)	4 (13)
Archives of Disease in Childhood	11	5 (45)	7 (64)	5 (45)	5 (45)
Archives of Disease in Childhood: Fetal & Neonatal Edition	1	0 (0)	1 (100)	0 (0)	1 (100)
Archives of Pediatric and Adolescent Medicine (JAMA Pediatrics)	6	2 (33)	3 (50)	3 (50)	0 (0)
Archives of Surgery (JAMA Surgery)	7	0 (0)	7 (100)	6 (86)	3 (43)
British Journal of Surgery	44	5 (11)	43 (98)	35 (80)	6 (14)
Circulation: Cardiovascular Interventions	3	0 (0)	3 (100)	1 (33)	0 (0)
Circulation: Heart Failure	5	2 (40)	5 (100)	3 (60)	0 (0)
Circulation Research	1	0 (0)	0 (0)	0 (0)	0 (0)
Circulation	7	2 (29)	6 (86)	4 (57)	0 (0)
Endoscopy	4	1 (25)	4 (100)	3 (75)	0 (0)
European Journal of Child and Adolescent Psychiatry	4	2 (50)	3 (75)	0 (0)	2 (50)
European Heart Journal	16	4 (25)	14 (88)	8 (50)	0 (0)
Journal of the American Academy of Child and Adolescent Psychiatry	7	2 (29)	4 (57)	2 (29)	0 (0)
Journal of the American College of Cardiology	15	1 (7)	14 (93)	6 (40)	0 (0)
Journal of the American College of Surgery	7	2 (29)	6 (86)	1 (14)	0 (0)
Journal of Neurology, Neurosurgery and Psychiatry	15	1 (7)	9 (60)	7 (47)	1 (7)
Journal of Pediatrics	9	4 (44)	7 (78)	5 (56)	3 (33)
JACC: Cardiovascular Imaging	2	1 (50)	2 (100)	1 (50)	0 (0)
JACCL Cardiovascular Interventions	2	0 (0)	2 (100)	2 (100)	0 (0)
Pediatric Allergy and Immunology	3	1 (33)	3 (100)	1 (33)	0 (0)
Pediatric Infectious Disease Journal	7	3 (43)	4 (57)	4 (57)	5 (71)
Pediatrics	30	14 (47)	29 (97)	14 (47)	10 (33)
Surgery for Obesity and Related Diseases	4	0 (0)	3 (75)	1 (25)	0 (0)

† One or more reproducible search was included. IS = Information Specialist

In the bivariate regression analyses, librarian or information specialist involvement and discipline (Pediatrics) were each significant predictors of the inclusion of a reproducible search strategy for at least one database. In the multivariable analysis, only discipline (Pediatrics) remained a significant predictor of inclusion of a reproducible search for at least one database. Discipline (Pediatrics) was also the only predictor in both the bivariate and multivariable analyses for including a reproducible search strategy for all databases in an article.

In the multivariable model predicting inclusion of at least one reproducible search, the Hosmer-Lemeshow goodness-of-fit test was non-significant (p = .603), the C-statistic was .731, collinearity was non-worrisome with tolerances ranging from .91-.99.

In the multivariable model predicting inclusion of a reproducible search for every database searched (if more than one database searched), the Hosmer-Lemeshow goodness-of-fit test was non-significant (p = .964), the C-statistic was .707, collinearity was non-worrisome with tolerances ranging from .91-.99.

## Discussion

In this article, we found that reporting of search strategies in systematic reviews published in 2012 was often poor, despite the increased visibility and awareness of reporting standards such as the PRISMA Statement and their use being required by some journals. Many core elements such as the date the search was executed, use of limits and inclusion of a full, Boolean search strategy were missing from the articles. Few articles (22%) included a reproducible search for at least one database and fewer (13%) included a reproducible strategy for all databases. There were significant differences between disciplines in how often different search elements were reported. Inclusion of a reproducible strategy was associated with discipline (Pediatrics) in both bivariate and multivariable analyses. Other potential predictors such as librarian/search specialist involvement, endorsement of a reporting guideline by the publishing journal, or mention of a reporting guideline in the article were not significant when discipline was included in the models.

One area in which this study expanded on previous studies was by looking at which search elements were reported and whether they were reported for specific databases in an article. For example, an article may indicate that three databases were searched from 1946–2012 using a specific set of medical subject headings (MeSH) and keywords. While on the surface this appears to constitute good reporting, a reader seeking to replicate or appraise the search may discover that while one of the three databases includes articles from 1946–2012, another one may cover 1981–2012. While one of the databases uses medical subject headings (MeSH) to index articles, another one may use a different controlled vocabulary or none at all. Very rarely can a single set of dates or search terms accurately describe a search in multiple databases. Providing a general search description such as this provides the illusion of good reporting.

In our study, we found a significant disconnect between general and specific reporting. While 91% of articles provided example search terms, only 25% of those that searched more than one database indicated that the search terms or full search strategy were for a specific database and only 14% provided a specific search or full search strategy for each database. Similarly, while approximately 70% of authors indicated a first or last date searched in a database, only 23% of those searching more than one database provided start/end dates for a specific database and 21% for all databases searched. This suggests that either education or more explicit guidelines are needed to help authors know what constitutes appropriate search reporting or what elements are required to make a reproducible search.

Less than a quarter of articles included a reproducible strategy according to our criteria (database searched, first and last years searched, if limits were applied, complete search strategy). Due to the differences in definitions of reproducibility, it is difficult to compare our rates with those from previous studies, though all share a finding of suboptimal reporting. Sampson et al. [29] examined non-Cochrane Collaboration systematic reviews published in 2004 and found that 15.5% contained a full, Boolean search strategy. Rethlefsen et al. [28] examined systematic reviews from General Medicine journals published in 2008–2012 and found that 44% contained a full Boolean strategy. Page et al [35] examined systematic reviews from February 2014 and found 45% contained a full Boolean search. In the current study, 33% of articles met this standard. Yoshii et al. [33] examined Cochrane Collaboration systematic reviews from 2006 and found that none included all of the elements then required by the Cochrane Collaboration. These requirements were very close to those that we used, with the exception that they required that the database provider be named. Maggio et al. [32] looked at medical education systematic reviews from 2009 and found that none were reproducible. They required that a Boolean search be present as well as the date the search was executed. Golder et al. [30] found that only 9% of searches in selected adverse effects systematic reviews were reproducible.

Just over a quarter of articles identified who designed or executed the search strategy. Systematic reviews require comprehensive searches, usually across multiple information sources. Knowing who designed the search and their background can help the reader evaluate how likely the search was to be comprehensive, especially if a reproducible search strategy is not included. Surprisingly, only 17% of articles mentioned the involvement of a librarian or information specialist, despite calls from the Institute of Medicine and other groups to include these search experts in systematic reviews [24–26]. Librarian involvement, however, may be underreported in published systematic reviews [28, 31].

Previous research found that librarian involvement in a systematic review was associated with higher quality searches that were more likely to be reproducible and use recommended search strategies [28, 31]. In the current study, librarian involvement was strongly associated with inclusion of a reproducible search in the bivariate regression analysis, but was no longer significant in the multivariable analysis after controlling for discipline. While the cause for this is unknown, our findings show that the disciplines most likely to include a librarian were also those most likely to include a reproducible search. Further research is needed to determine the impact of librarians on systematic review search quality independent of disciplinary behavior.

Neither journal endorsement of a reporting guideline nor mention of the reporting guideline in the article text was associated with inclusion of a reproducible search. Surprisingly, included articles from Surgery journals referenced reporting guidelines more often than those from Pediatrics or Cardiology journals, but were less likely to report reproducible searches. This mirrors the findings of the review by Stevens et al. [22] which found that PRISMA scores did not significantly differ between journals that endorsed PRISMA and those that did not or between the same journal before or after PRISMA was endorsed. However, this contradicts Page et al. [35] which found that many reporting items were likely to be found if PRISMA was mentioned. Though they found overall reporting characteristics to favor those mentioning PRISMA, Page et al. did not find any significant difference on reporting start and end years of search or inclusion of a full Boolean search, similar to this study. They did find significant differences in reporting these two items between Cochrane and non-Cochrane reviews. Only two of the studies we identified were abridged or modified versions of Cochrane reviews [40,41], so no comparisons could be made between Cochrane and non-Cochrane reviews in our sample. It is important to note that Page et al. found lower rates than we did for both mention of a reporting guideline (29% vs. 55%) and inclusion of a PRISMA-style flowchart (69% vs. 89%). These were the largest differences between our studies in terms of search element reporting and may reflect our focus on high-impact journals versus their focus on all systematic reviews published in a single month. Further efforts may be necessary to ensure that authors who indicate they are following a reporting guideline actually are. One potential solution could be having librarians or information specialists peer-review search strategies of submitted systematic reviews similar to the role of a statistician peer-reviewer.

In our multivariable analyses, articles from Pediatrics journals were significantly more likely to include reproducible searches than those from Surgery. There were also significant disciplinary differences in the reporting of several search elements and procedures such as dates searched. It is unclear what the specific cause of these differences may be. One explanation of this could be that authors tend to mirror what they are reading when they are writing. If an author sees that systematic reviews in their discipline tend to include certain elements, such as a reproducible search, they may assume it is best practice and include it as well. It is also possible that editors or peer-reviewers of Pediatrics journals are more stringent in requiring that authors follow reporting guidelines. Further studies are needed to confirm and determine the cause of the observed difference.

There are several limitations that temper the results of this paper. First, all articles examined come from 2012. It is possible that as reporting guidelines such as PRISMA and conduct guidelines such as those from the Cochrane Collaboration and Institute of Medicine have become more well-known and reporting quality has improved in recent years. Comparisons with Page et al.’s findings [35] from 2014 publications indicate similar trends in poor reporting and search characteristics, however. Moreover, when comparing search strategy characteristics between systematic reviews written in 2004 and 2014, Page et al. found only small improvements on variables such as inclusion of a Boolean search strategy. This suggests the rates of improvement are slow and there are unlikely to be meaningful differences between systematic reviews published in 2012 and the following years. Future research is needed to track how reporting of search strategies my change in the future.

Second, the criteria for a reproducible search were based on existing reporting guidelines, but also on the authors’ experiences and opinion. The criteria chosen, however, were generous and slightly less restrictive than those proposed by the Cochrane Collaboration, have been used in previous research, and were designed to reflect real-world reproducibility. Nevertheless, criteria were subjective and further work is required to create better guidelines on what makes a reproducible search. Finally, we examined systematic reviews in only 3 disciplines and only in high-impact journals within those disciplines. While our results reflect those of earlier studies, future research is needed to see whether the same pattern of poor reporting holds in other specialties and lower impact journals.

## Conclusion

Based on the results of our study, it is clear many authors are still reporting incomplete or non-reproducible searches. Simply creating reporting guidelines is meaningless unless their use is reinforced and encouraged. The finding that most authors are reporting a single strategy that covers all databases suggests that authors are unaware of what should be reported and how. While disciplinary culture may reinforce the use of reproducible searches, journal editors and peer-reviewers should help authors ensure that they are reporting full, reproducible search strategies. Librarians and other search specialists working with authors need to recommend full reporting and follow-up to make sure it happens. Finally, better reporting guidelines for search strategies need to be developed to make it easier for editors, peer-reviewers and authors to know what should be reported and ensure more transparent systematic reviews in the future.

## Supporting Information

S1 Data Extracted search strategies. Contains the raw data extracted from each article by the authors. Field descriptions and coded values are at the bottom of the file.
(CSV)

S2 Data Analyzed search strategies. Raw data were summarized and enriched to indicate if an item was reported for any database vs. all databases and add additional elements. These are the data that underlie the analyses in the paper. Field descriptions and coded values are at the bottom of the file.
(CSV)

S1 Table Search Reporting Elements Used in All Databases. Examines how often each search element was reported for all databases within a given articles. Limited to those articles which searched more than one database.
(PDF)
