# Supplementary material for: Reproducibility of Search Strategies Is Poor in Systematic Reviews Published in High-Impact Pediatrics, Cardiology and Surgery Journals: A Cross-Sectional Study
Source: PLoS One. 2016 Sep 26;11(9):e0163309. doi: 10.1371/journal.pone.0163309 (PMC5036875; doi:10.1371/journal.pone.0163309)
Supplement: S1 Table — Examines how often each search element was reported for all databases within a given articles. Limited to those articles which searched more than one database. (PDF) [file pone.0163309.s003.pdf]

S3 Table: Reporting of Search Strategy Elements For All Included Databases *% (n)*

	Surgery	Pediatrics	Cardiology	Total	
**Core Search Elements**					
Named Database Provider	4 *(5)*	10 *(7)*	2 *(1)*	6 *(13)*	
Named Database Provider or Provider Could Be Assumed	4 *(5)*	12 *(8)*	4 *(2)*	7 *(15)*	
Specific Year Given for First Date Searched	50 *(60)*	55 *(37)*	31 *(14)*	48 *(111)*	‡*
Specific Date Given for Last Date Searched ✦	65 *(78)*	63 *(43)*	64 *(29)*	65 *(150)*	
Indicated Date Search Was Executed	24 *(28)*	14 *(21)*	16 *(7)*	21 *(49)*	
Indicated Date Search Was Updated	1 *(1)*	4 *(3)*	0 *(0)*	2 *(4)*	
Provided Specific Search Terms	87 *(103)*	75 *(51)*	82 *(37)*	82 *(191)*	
Provided a Full Search Strategy	13 *(15)*	46 *(31)*	22 *(10)*	24 *(56)*	†*** ‡* §*

Limited to articles searching more than one database. † Pediatrics vs Surgery ‡ Pediatrics vs Cardiology § Surgery vs Cardiology. *p < .05, ** p < .01, ***p < .001. ✦ Specific month and year.
